# Increasing Plant Based Foods or Dairy Foods Differentially Affects Nutrient Intakes: Dietary Scenarios Using NHANES 2007–2010

**DOI:** 10.3390/nu8070422

**Published:** 2016-07-11

**Authors:** Christopher J. Cifelli, Jenny A. Houchins, Elieke Demmer, Victor L. Fulgoni

**Affiliations:** 1 National Dairy Council, 10255 West Higgins Road, Suite 900, Rosemont, IL 60018-5616, USA; jenny.houchins@dairy.org (J.A.H.); elieke.demmer@dairy.org (E.D.); 2 Nutrition Impact, LLC, 9725 D Drive North, Battle Creek, MI 49014, USA; vic3rd@aol.com

**Keywords:** plant-based foods, dairy, nutrient adequacy, sustainability

## Abstract

Diets rich in plant foods and lower in animal-based products have garnered increased attention among researchers, dietitians and health professionals in recent years for their potential to, not only improve health, but also to lessen the environmental impact. However, the potential effects of increasing plant-based foods at the expense of animal-based foods on macro- and micronutrient nutrient adequacy in the U.S. diet is unknown. In addition, dairy foods are consistently under consumed, thus the impact of increased dairy on nutrient adequacy is important to measure. Accordingly, the objective of this study was to use national survey data to model three different dietary scenarios to assess the effects of increasing plant-based foods or dairy foods on macronutrient intake and nutrient adequacy. Data from the National Health and Nutrition Examination Survey (NHANES) 2007–2010 for persons two years and older (*n* = 17,387) were used in all the analyses. Comparisons were made of usual intake of macronutrients and shortfall nutrients of three dietary scenarios that increased intakes by 100%: (i) plant-based foods; (ii) protein-rich plant-based foods (i.e., legumes, nuts, seeds, soy); and (iii) milk, cheese and yogurt. Scenarios (i) and (ii) had commensurate reductions in animal product intake. In both children (2–18 years) and adults (≥19 years), the percent not meeting the Estimated Average Requirement (EAR) decreased for vitamin C, magnesium, vitamin E, folate and iron when plant-based foods were increased. However the percent not meeting the EAR increased for calcium, protein, vitamin A, and vitamin D in this scenario. Doubling protein-rich plant-based foods had no effect on nutrient intake because they were consumed in very low quantities in the baseline diet. The dairy model reduced the percent not meeting the EAR for calcium, vitamin A, vitamin D, magnesium, and protein, while sodium and saturated fat levels increased. Our modeling shows that increasing plant-based foods could lead to unintended dietary outcomes without simultaneous changes in the types and amounts of plant foods currently consumed. Increasing dairy foods, which are currently under-consumed, could assist in improving the intakes of many nutrients of concern.

## 1. Introduction

The current diet in the U.S., as in other Western countries, is characterized by a high intake of foods of animal origin [1,2]. Globalization and increasing wealth in developing nations has increased the demand of meat and dairy products in those regions [1,3,4]. These trends have raised issues regarding the strain on the earth’s non-renewable natural resources such as land, water, and fossil fuels [1,2,5,6,7,8,9]. There is also increasing concern about climate change, greenhouse gas emissions (GHGEs), deforestation, and loss of biodiversity [1,2,4,5]. Coupled with these concerns is the fact that while the Earth has the capacity currently to feed seven billion people, it is estimated that the world’s population will reach nine billion by 2050 [10]. Recognition of these factors has contributed to increased research on optimizing dietary patterns to minimize environmental footprints. For instance, Hedenus et al. reported that reduced meat and dairy intake were indispensable to meet the European Union (EU) 2050 climate targets [11]. Similarly, Bryngelsson et al. concluded that reductions in ruminant meat are unavoidable if the EU targets are to be met [12].

The Food and Agriculture Organization (FAO) has defined “sustainable diets” as those that meet nutritional, environmental, affordability and cultural criteria [4]. Although several studies have assessed the potential impact of decreasing animal foods and increasing plant food on GHGEs and land use, there are limited data on their potential effect on nutritional intake. One study showed that if 25%–50% of animal-derived foods were replaced with plant-based foods, there would be a 40% reduction in nitrogen emissions, 25%–40% reduction in GHGE, 23% less land use and a 40% reduction in saturated fat intake [2]. Along those lines, a recent working paper published by the World Resources Institute reported that diet shifts that reduce protein consumption from animal-based foods could help close the estimated food gap that is proposed to occur in 2050 [13]. However, as described by Gustafson et al., improving dietary diversity and micronutrient adequacy are more important than simply ensuring adequate protein and calories for ensuring sustainable nutrition security [14]. Decreasing the consumption of certain foods of animal origin solely for environmental purposes may have unintended consequences nutritionally. For instance, dairy products are a source of key vitamins and minerals [15], including nutrients of public health concern-calcium, vitamin D, and potassium but the majority of Americans consume less dairy servings than recommended by the 2015 Dietary Guidelines for Americans (DGA) [15,16]. It has been noted that few people achieve their recommended intake of several shortfall nutrients without consuming the recommended amounts of dairy foods [15]. Thus, while dairy production does impact the environment, the environmental cost needs to be examined in conjunction with the nutritional benefits dairy provides.

The objective of this study was to use national survey data to model different dietary scenarios to assess the potential effects of increasing plant-based foods (and concomitantly decreasing animal foods) or dairy foods on macronutrient intake and nutrient adequacy. To accomplish this goal, we conducted diet modeling exercises based on the following: (1) increasing the amount of plant-based foods by 100% based on current consumption patterns while decreasing animal product consumption; (2) increasing the amount of specific protein-rich plant foods (i.e., legumes, nuts, seeds, soy) by 100% based on current consumption patterns while decreasing animal product consumption; and [3] increasing the amount of milk, cheese, and yogurt as consumed by 100%. We hypothesized that micronutrient adequacy would improve in each scenario, but that the greatest improvements in nutrients of public health concern would be observed when dairy foods were increased.

## 2. Materials and Methods

Data from What We Eat in America (WWEIA), which is the dietary component of the National Health and Nutrition Examination Survey (NHANES), 2007–2010 for persons 2 years or older excluding those pregnant/lactating were used in these analyses. NHANES is a nationally representative cross-sectional study that utilizes a stratified, multistage probability sample of the non-institutionalized U.S. population [17]. Food and nutrient intake were determined from two nonconsecutive 24-h dietary recalls. The first was conducted by in-person interview at the Mobile Examination Center and the second was obtained via a telephone interview a few days later. Only data coded as reliable for those 2+ years of age were used (*n* = 17,387). The National Cancer Institute (NCI) method was used for estimating usual intakes and the covariates used were recall day sequence, Dietary Reference Intakes (DRI) age group and recall weekday/weekend indicator [18]. The cut-point method was used to assess nutrient inadequacy (percentage of the population below the estimated average requirement (EAR) for all nutrients examined that had an EAR except that the probability method was used for assessing iron inadequacy [19]. For nutrients with an adequate intake (AI), we assessed the percentage of the population above the AI. The nutrients examined in this study were designated as underconsumed or overconsumed by the 2015 Dietary Guidelines Advisory Committee [20]. All analyses were performed for gender combined.

In this study, three dietary scenarios were examined in children (2–18 years) and adults (19+ years) and compared to the baseline, usual diet. In Scenario 1, currently consumed plant-based foods were increased by 100% with commensurate decrease in animal products on a gram per gram basis. This approach illustrates a potential outcome if consumers were to modify their diet based on what they currently consume, even though these foods may not be the most nutrient dense options, because major changes in dietary habits are typically difficult to change. In Scenario 2, currently consumed protein-rich plant-based foods (i.e., beans, peas, legumes, nuts, seeds, and soy products), were increased by 100%, with commensurate decrease in animal products on a gram per gram basis. Since it was anticipated that protein intake would decrease when plant foods as currently consumed were increased and animal products were reduced, this scenario specifically increased higher protein plant foods. Furthermore, a key message from the Institute of Medicine workshop on healthy sustainable diets was that plant-based protein sources were worth considering as an alternative to animal-based protein sources [5]. Scenarios 1 and 2 were calorically matched to the baseline diet to model increases in plant foods while maintaining caloric balance. For Scenario 3, currently consumed milk, cheese, and yogurt were increased by 100%, using the United States Department of Agriculture (USDA) dairy composite nutrient profile for the increased dairy products [21]. Dietary guidelines in the United States and elsewhere recommend 2–4 servings of dairy products per day for optimal nutrition [22,23,24]; therefore, this scenario increased dairy product levels by 100% without any reduction in plant or other food intake. The outcome is a model aligned with recommended amounts of dairy product intake. Mixed dishes containing either dairy products (e.g., macaroni and cheese) or plant foods higher in protein (e.g., bean soup) were not increased because of the difficulty associated with isolating the individual component of interest, and its associated nutrients, from the other foods contained within the mixed dish.

The Food Pattern Equivalent Database for each NHANES release was used to define various types of foods in the analyses [25,26]. Plant-based foods were defined as food codes with zero servings of dairy, meat/poultry/fish, and eggs with non-zero servings for the sum of servings of fruits, vegetables, legumes, grains, soy products and nuts/seeds. Animal-based foods were defined as food codes with zero servings of the sum of servings of fruits, vegetables, legumes, grains, soy products and nuts/seeds with non-zero servings for the sum of dairy, meat/poultry/fish and eggs. Higher protein-based foods were defined using USDA food category “Plant-based Protein Foods”. This category includes “Beans, peas and legumes”, “Nuts and seeds”, and “Processed Soy Products” [27]. Nutrient composition of “dairy” was based on the dairy food composite used by USDA for dietary pattern analyses [21]. Adequate protein intake was based on g protein/kg body weight (BW) and g/kg ideal BW as recommend by DRI [28].

### Statistical Analysis

SAS 9.2 (SAS Institute; Cary, NC, USA) and SUDAAN 11 (Research Triangle Institute; Research Triangle Park, NC, USA) were used for all analyses. NHANES survey weights, strata, and primary sampling units were used in all calculations. The National Cancer Institute method was used to estimate usual intakes of the food groups and selected nutrients to assess the percentage of the population meeting nutrient adequacy [18,29]. Given that we know there are differences in modeling scenarios by design, typical statistical testing was not appropriate. Thus, to assess meaningful differences in the modeling scenarios we examined means and 95th percentile confidence limits of the means. When there were non-overlapping confidence intervals we deemed the difference as being meaningful. This approach has been utilized previously in dietary pattern studies [30].

## 3. Results

Table 1 and Table 2 show the changes in the various food groups associated with each scenario for children and adults, respectively. Changes in individual food group intakes were based on each food as consumed and did not account for those foods in mixed dishes. As such, there was range of increases and decreases, which reflects differences in how each food is consumed. Total fruit, total grains, total vegetables, legumes, nuts and seeds, and soy were all increased while eggs, meat, poultry and fish, and dairy were decreased in Scenario 1 that increased plant-based foods as consumed for both age groups. In Scenario 2, legumes, nuts and seeds, and soy were increased with no other change in plant-based foods. The increase in these foods was slightly less than double because other mixed dishes that contained some legumes, nuts and seeds, and soy were not increased. The magnitude of the decrease in animal-based foods was lower in this model as compared to the plant-as-consumed model due to the very low intakes of legumes, nuts and seeds, and soy at baseline. Dairy product consumption, in Scenario 3, was nearly doubled without any corresponding change in plant-based foods.

### 3.1. Children and Adolescents (2–18 Years)

Shifts in macronutrients, nutrients of public health concern and shortfall nutrients specified by the DGA [22] are shown in Table 3 and Table 4 for each of the scenarios for this population group. Increasing plant foods as currently consumed (Scenario 1) resulted in an increase in energy, carbohydrate, added sugars and dietary fiber intake and a decrease in protein, total fat and saturated fat intake. In this scenario, the percentage of children and adolescents that exceeded the AI for dietary fiber increased dramatically, but those not meeting the EAR for protein almost tripled versus baseline levels. In contrast, Scenario 2 which increased protein-rich plant foods did not impact any of the macronutrients. When currently consumed dairy foods were increased (Scenario 3), intakes of energy, carbohydrate, protein, total fat, saturated fat and added sugar were higher than baseline intake values, as expected. Protein intake was highest with this scenario compared to the usual diet and the other dietary scenarios with the percent of children and adolescents falling below the EAR for protein at 1% or lower.

Compared to baseline, increasing the consumption of plant-based foods as consumed (Scenario 1) enhanced the mean intakes of vitamins C and E, folate, magnesium and iron resulting in improvements in nutrient adequacy for these vitamins and minerals. The greatest changes were noted for iron with a reduction of 50% and for vitamin C with a reduction of 43% in those not meeting EAR. However, the percentage of children and adolescents not meeting the EAR increased for vitamin A, vitamin D, and calcium. In the case of calcium, the percentage not meeting the EAR increased by 50%. There were no differences between the baseline diet and the increased plant protein-rich food scenario (Scenario 2) in mean nutrient intake or in the percentages below the EAR or above the AI. Increasing dairy food intake (Scenario 3), improved mean intakes of vitamin A, vitamin D, magnesium, calcium and potassium in relation to the baseline diet. The percent of children and adolescents not meeting the EAR decreased for these vitamins and minerals with decreases of 33% for vitamin D and 55% for calcium. Intakes of potassium and sodium were also increased in the dairy model.

### 3.2. Adults (19+ Years)

Table 5 and Table 6 report the usual intake of macronutrients and nutrients of public health concern and shortfall nutrients in adults 19 years or older. An increase in the intake of plant-based foods that were currently consumed (Scenario 1) raised carbohydrate and dietary fiber intake, whereas intakes of protein, total fat and saturated fat were lower than baseline levels. The percentage of adults with dietary fiber intakes above the AI increased 267% compared to baseline, whereas the proportion of adults with protein intakes below the EAR increased 321%, exceeding 15% of the population. There were no differences in macronutrient intake between the usual diet and the dietary scenario that increased protein-rich plant foods (Scenario 2). Mean intakes for energy, carbohydrate, fat, saturated fat and added sugars were greater in Scenario 3, which doubled dairy foods based on current consumption patterns as compared to the usual diet. As observed in children and adolescents, protein intake was the highest in the dairy scenario compared to baseline and other dietary scenarios. This is reflected in the percentage of the adult population not meeting the EAR for protein, which was the lowest in the dairy scenario.

Mean intakes increased and the percentage of adults below the EAR decreased for vitamin C, vitamin E, folate, magnesium and iron when plant-based foods that were currently consumed were increased (Scenario 1) compared to the baseline diet. The largest decrease of the percentage of adults below the EAR was observed for folate, followed by vitamin C. Mean sodium intake also decreased, with a reduction of 162 mg. However, within the same scenario, the percentage of adults not meeting the EAR increased for vitamin A, vitamin D, and calcium, with calcium faring the worst. Doubling the protein-rich plant-based foods (Scenario 2) increased vitamin E and magnesium intake compared to the usual diet and lowered the percent of the adult population below the EAR for vitamin E. Increasing dairy foods (Scenario 3) enhanced the mean intake of vitamin A, vitamin D, magnesium, and calcium. The percentage of adults below the EAR was markedly reduced for vitamin A, vitamin D, magnesium and calcium in this model. Reductions in those adults below the EAR were greatest for calcium (48%) and for vitamin A (34%). Potassium consumption was also greatly improved with a 183% increase in the proportion of the adult population with intakes above the AI in the dairy scenario compared to baseline. However, mean sodium intake increased by 244 mg in the dairy scenario.

## 4. Discussion

Recent estimates suggest that the population of the world will reach nine billion by 2050 and providing sufficient nutritious food is expected to be a challenge for the global food industry [10,31]. Current food production practices and consumption patterns are considered to be unsustainable based on the strain on the Earth’s natural resources and the negative impact on the environment [10,31]. A significant share of the resource utilization and environmental burden is attributed to meat and dairy food production compared to plant food production [1,6,7,8]. As a means of addressing these concerns, numerous studies have begun recommending increased consumption of plant-based foods and reduced intake of animal products to mitigate environmental impacts [11,12]. Further, Public Health England has recently recommended diets high in plant-based products and lower in meat and dairy to improve health and limit environmental footprints [32]. However, the potential effects of increasing plant-based foods at the expense of animal-based products on macronutrient intakes and micronutrient nutrient adequacy remains unknown. The dietary scenarios presented in this study contribute to the growing discussion on the intersection of nutrition and sustainability by showing that recommendations to change the diet to reduce the environmental impact should consider all potential nutritional impacts as well.

Our results showed that increasing or decreasing certain foods within current consumption patterns in the diet results in profound effects on nutrient adequacy. For instance, increasing consumption of plant-based foods currently consumed in the U.S. resulted in decreased intakes of total fat, saturated fat, and protein, while increasing dietary fiber in both children and adults. Further, increasing fruits, vegetables, and grain-based products in the plant-based model improved intakes of several nutrients in both children and adults. However, in both children and adults, increasing plant-based foods resulted in an increase of the percentage of individuals not meeting the EAR for vitamin A and two nutrients specified in the 2015 DGA as nutrients of public health concern, namely calcium and vitamin D [22]. Our data highlight that indiscriminate recommendations to increase plant-based foods may adversely affect nutrient intakes when substituted for certain nutrient-dense animal foods. Thus, future research studies on the interplay between dietary choices and environmental impact should consider macronutrient and micronutrient adequacy, as well as bioavailability of nutrients in different foods, to ensure proposed diets meet nutrient needs. Furthermore, recommendations from the public health community need to continue to emphasize the types and amounts of grains, vegetables, and fruits to be consumed to ensure that nutrient gaps are closed.

Interestingly, in our modeling, increasing the intake of high-protein plant-based foods did not appreciably alter macronutrient intakes or nutrient adequacy in children. In adults, similar results were observed with the exception that intakes of vitamin E and magnesium improved. This finding was due in part to the current very low consumption of these foods by the U.S. population, and therefore doubling intake may not have increased the consumption of these foods enough to observe meaningful changes in nutrient intake. Results of this scenario suggest greater changes (i.e., more than 100% increase) will be needed to obtain substantial changes in nutrient intake with these types of foods. Future work is needed examine the nutritional and environmental impact of even greater increases in higher protein plant-based foods than used in our study. In addition, research in this area should consider differences in protein quality that exist between animal- and plant-based foods as protein quality is important for muscle growth and function throughout the lifespan [33]. Further, behavioral research is needed to better understand if actually achieving higher intakes would be culturally acceptable and sustainable over time.

Doubling dairy product intake based on current consumption patterns enhanced mean intakes of vitamin A, vitamin D, magnesium, calcium, and potassium in both children and adults. The percentage of children and adults below the EAR was markedly reduced for all five of these nutrients. Thus, given this scenario was close to dairy recommendations, our data support that increased dairy consumption can help improve nutrient adequacy especially for most nutrients of public health concern [22]. However, the dairy scenario resulted in increased intakes of nutrients in both children and adults that have been inversely associated with health, including excess energy, saturated fat, and sodium. Given the current obesity epidemic, the increase in energy intake is a potential concern. However, there is a large body of evidence indicating that dairy consumption is not associated with weight gain, and adequate dairy consumption my benefit weight loss during energy restriction, at least in studies lasting one year or less [34,35,36]. These data suggest that recommended amounts of dairy can be incorporated into a healthy eating pattern. Along similar lines, any increase in saturated fat intake can be a cause for concern due to the link between saturated fat intake and increased cardiovascular disease risk [37]. However, the understanding of how different fatty acids effect development of cardiovascular disease risk continues to evolve. For example, a recent systematic review and meta-analysis reported that the available evidence does not support recommendations to consume more polyunsaturated fatty acids and less saturated fatty acids [38]. Specific to dairy foods, several recent meta-analyses [39,40] and systematic reviews [41,42,43,44] have either found no association or an inverse association between the consumption of dairy foods and cardiovascular risk. Finally, higher sodium intakes are associated with elevated blood pressure and increased risk for hypertension, especially in at-risk individuals. Several recent studies have reported a U-shaped association between sodium and cardiovascular disease events, suggesting that low sodium intakes may not be healthy for all individuals [45,46]. Additionally, while sodium levels were increased in both dairy scenarios, intakes of potassium were also meaningfully increased. Thus, the sodium-to-potassium ratio was actually lower in dairy scenarios as compared to the baseline model. Given the relationship between sodium-to-potassium ratio and hypertension risk, the improvement in the ratio would suggest that dairy may actually improve vascular health, or at the very least, impart neutral effects. Indeed, several meta-analyses [47,48] and a recent randomized clinical trial [49] support this relationship. Taken together, the results of these analyses illustrate the importance of considering the complete nutrient package of all foods before making recommendations to increase or decrease consumption of certain foods for environmental purposes.

Additional studies are needed to fully understand the significance of increasing specific types of plant-based foods on diet quality and overall health, as well as the impact these dietary changes will have on the environment. It has been assumed that a shift towards healthy diets that are mainly plant-based will have a favorable impact on the environment because plant-based foods have lower GHGEs per unit weight than do animal foods [50]. It has further been assumed that diets with the lower GHGEs are the healthiest [7]. For example, Springmann et al. reported that transitioning to plant-based diets could reduce global mortality and GHGE, as well as provide an overall economic benefit [51]. However, this has not always been observed, particularly when GHGEs are assessed on a per calorie basis. An analysis of the GHGE values for 483 foods from five major food groups revealed that grains and sweets with low nutrient content and high energy density had the lowest GHGEs per 100 g and per 100 kcal [52]. Frozen and processed fruits and vegetables had very low GHGEs per 100 g, but because of their low energy densities, had higher GHGEs when expressed per 100 kcal [52]. In addition, a French study noted that diets containing large amounts of plant-foods at a given level of energy were not those with the lowest GHGEs [53]. These researchers suggest that fruit and vegetables appear to have GHGEs similar to those of animal products when expressed on a per calorie basis because they need to be eaten in larger quantities to meet energy requirements. Finally, a recent systematic review concluded that diet scenarios with lower GHGE compared with usual dietary patterns may not result in improvements in nutritional quality due to high sugar consumption and lower micronutrient intakes [54]. The results of the above studies illustrate the need for a holistic approach that simultaneously examines the nutritional, environmental, economic, and social impacts of changes to dietary consumption. For example, Gustafson et al. defined seven metrics that should be considered when evaluating the impact of changes to the food system to sustainability and human nutrition outcomes [14]. In this manner, we can begin to formulate a more complete understanding of how altering dietary intakes will impact food security and the environment as a whole.

Dairy products are an important source of many essential vitamins and minerals in the U.S. diet. At current intakes of about two servings/day, the dairy group (milk, cheese and yogurt) contributes 51% of the calcium, 58% of the vitamin D, 28% of the phosphorus, 28% of the vitamin A, 26% of the vitamin B12, 25% of the riboflavin, 18% of the protein, 16% of the zinc, 16% of the potassium and 13% of the magnesium, on average, in exchange for only 10% of the daily caloric intake, 14% of total fat, 26% saturated fat, and 11% of sodium for persons ≥2 years [55]. However, there is concern that dairy production has a negative environmental impact, in part due to high outputs of GHGEs. In the U.S. it is currently estimated that the dairy sector contributes 1.9% of total GHGEs [56]. The U.S. dairy industry is moving towards the implementation of several strategies throughout the dairy supply chain in order to meet 2020 goals of a 25% reduction in GHGEs [56]. Moreover, a recent study examined the effects of one additional serving of milk per day on nutrition- and environmental-related disability-adjusted life years (DALYs) and found an overall reduction in DALYs [57]. The increase in milk in the DALYs analysis is similar to the increase observed in our modeling exercise, suggesting that the environmental impact of increased dairy might be offset by improved health benefits. Thus, while dairy products have higher GHGE values per 100 g as compared to other foods [52], environmental impact needs to be assessed in conjunction with nutrient intake, nutrient adequacy and overall contribution to health.

Strengths of this study include the use of a large, nationally representative database for determining the nutritional impact of changing plant and dairy products; the use of the Food Pattern Equivalent Database to develop the diet scenarios; use of usual intake methodology to assess nutrient adequacy, and modeling dietary changes that would be practical for most people. Limitations of the study include the use of self-reported intake data which may over- or under-represent actual intake especially in overweight and obese individuals. Additionally, our data were limited to the foods available in NHANES 2007–2010 which may not accurately represent all foods or product formulations currently in the marketplace. Further, mixed dishes that contained dairy and plant foods were not increased in our models. This could contribute to an underestimation and/or overestimation of nutrient intakes in the different scenarios. Additionally, we did not include a model where other animal-based foods were increased in this study. Finally, we combined age and gender groups in this study, thus it is possible that certain results may vary for specific age/sex groups.

## 5. Conclusions

Overall, the results of this modelling study show that indiscriminate recommendations to increase plant-foods could lead to the improvements in some nutrients but also lead to unintended dietary outcomes without behavior change. An important component of sustainable diets according to FAO is cultural acceptability [4]. With the message of increasing plant-based foods, there is the possibility that people will not increase their intake of protein-rich and other nutrient-dense plant foods as recommended, but instead increase the consumption of less healthy plant-based foods currently being consumed, while eschewing dairy and other nutrient-rich animal products. As an example, recommendations over the past decade to decrease fat intake did not lead to more fruit, vegetable and whole grain intake, but led to an increase of refined grain and sugar consumption that appears have contributed, at least in part, to the obesity epidemic [58]. Specific recommendations to increase low fat and nonfat dairy foods in conjunction to increasing healthy plant-based foods will help to close some of the nutrient gaps currently present among Americans of all ages. Our results reinforce the well-balanced diet advocated by the 2015–2020 Dietary Guidelines for Americans for meeting nutrient adequacy and health.

## Figures and Tables

**Table 1:** Usual food group intake in children, 2–18 years (National Health and Nutrition Examination Survey (NHANES) 2007–2010) compared to a 100% increase in plant foods, 100% increase in protein-rich plant foods, and 100% increase in dairy foods ^1,2^.

	Usual Diet (Baseline)	100% Increase in Plant Foods (Scenario 1)	100% Increase in Protein-Rich Foods (Scenario 2)	100% Increase in Dairy Foods (Scenario 3)
Egg (oz eq)	0.35 ± 0.02	0.18 ± 0.01	0.34 ± 0.02	0.35 ± 0.02
Legumes (cup eq)	0.06 ± 0.01	0.08 ± 0.01	0.09 ± 0.01	0.06 ± 0.01
Meat, poultry, fish (oz eq)	3.40 ± 0.1	2.1 ± 0.1	3.4 ± 0.1	3.4 ± 0.1
Nuts and seeds (oz eq)	0.36 ± 0.03	0.55 ± 0.04	0.61 ± 0.1	0.36 ± 0.03
Soy (oz eq)	0.03 ± 0.00	0.04 ± 0.01	0.04 ± 0.00	0.03 ± 0.00
Total dairy (cup eq)	2.2 ± 0.0	1.1 ± 0.0	2.1 ± 0.0	3.7 ± 0.1
Total fruit (cup eq)	1.1 ± 0.0	1.7 ± 0.1	1.1 ± 0.0	1.1 ± 0.0
Total grain (oz eq)	6.5 ± 0.1	8.6 ± 0.1	6.5 ± 0.1	6.5 ± 0.1
Total vegetable (cup eq)	0.90 ± 0.03	1.3 ± 0.0	0.91 ± 0.03	0.90 ± 0.03

^1^ Each of the modeling exercises were based on plant-based foods, protein-rich plant-based foods, and dairy foods as consumed. The models increased/decreased foods as consumed and did not change the individual foods found in mixed dishes; ^2^ Values are mean ± standard error.

**Table 2:** Usual food group intake in adults, 19+ years (NHANES 2007–2010) compared to a 100% increase in plant foods, 100% increase in protein-rich plant foods, and 100% increase in dairy foods ^1,2^.

	Usual Diet (Baseline)	100% Increase in Plant Foods (Scenario 1)	100% Increase in Protein-Rich Foods (Scenario 2)	100% Increase in Dairy Foods (Scenario 3)
Egg (oz eq)	0.52 ± 0.02	0.25 ± 0.01	0.49 ± 0.01	0.52 ± 0.02
Legumes (cup eq)	0.11 ± 0.01	0.15 ± 0.01	0.17 ± 0.01	0.11 ± 0.01
Meat, poultry, fish (oz eq)	4.9 ± 0.1	2.8 ± 0.1	4.8 ± 0.1	4.9 ± 0.1
Nuts and seeds (oz eq)	0.63 ± 0.03	0.90 ± 0.04	1.1 ± 0.1	0.63 ± 0.03
Soy (oz eq)	0.08 ± 0.01	0.11 ± 0.01	0.10 ± 0.01	0.08 ± 0.01
Total dairy (cup eq)	1.7 ± 0.0	0.81 ± 0.02	1.6 ± 0.0	2.8 ± 0.1
Total fruit (cup eq)	1.1 ± 0.0	1.5 ± 0.0	1.1 ± 0.0	1.7 ± 0.03
Total grain (oz eq)	6.5 ± 0.1	8.4 ± 0.1	6.5 ± 0.1	6.5 ± 0.1
Total vegetable (cup eq)	1.6 ± 0.0	2.1 ± 0.0	1.6 ± 0.03	1.6 ± 0.03

^1^ Each of the modeling exercises were based on plant-based foods, protein-rich plant-based foods, and dairy foods as consumed. The models increased/decreased foods as consumed and did not change the individual foods found in mixed dishes; ^2^ Values are mean ± standard error.

**Table 3:** Usual macronutrient intake in children, 2–18 years (NHANES 2007–2010) compared to a 100% increase in plant foods, 100% increase in protein-rich plant foods, and 100% increase in dairy foods ^1,2^.

	Usual Diet (Baseline)	100% Increase in Plant Foods (Scenario 1)	100% Increase in Protein-Rich Plant foods (Scenario 2)	100% Increase in Dairy Foods (Scenario 3)
Energy (kcal)	1920 ± 16	2028 ± 15 *	1939 ± 15	2183 ± 17 *
Carbohydrate (g)	256 ± 2	307 ± 2 *	258 ± 2	283 ± 2 *
% Kcal from carbohydrate	53.2 ± 0.2	60.6 ± 0.2 *	53.0 ± 0.2	51.7 ± 0.1 *
Protein (g)	68.7 ± 0.6	58.0 ± 0.6 *	69.3 ± 0.6	82.5 ± 0.7 *
% Kcal from protein	14.3 ± 0.1	11.3 ± 0.1 *	14.3 ± 0.1	15.1 ± 0.1 *
Total fat (g)	71.3 ± 0.6	66.5 ± 0.7 *	73.1 ± 0.7	83.0 ± 0.7 *
% Kcal from fat	32.2 ± 0.1	27.9 ± 0.2	32.4 ± 0.2	32.9 ± 0.2 *
Saturated fat (g)	25.2 ± 0.2	20.1 ± 0.2 *	25.3 ± 0.2	32.3 ± 0.3 *
% Kcal from saturated fat	11.2 ± 0.1	8.4 ± 0.1 *	11.3 ± 0.1	12.6 ± 0.1 *
Dietary fiber (g)	13.5 ± 0.2	18.2 ± 0.2 *	14.2 ± 0.2	14.0 ± 0.2
Added sugars (tsp eq)	19.7 ± 0.3	21.1 ± 0.3 *	19.6 ± 0.3	21.8 ± 0.3 *

^1^ Each of the modeling exercises were based on plant-based foods, protein-rich plant-based foods, and dairy foods as consumed; ^2^ Values are usual intake ± standard error; * Meaningfully different from baseline due to non-overlapping 95th percentile confidence intervals.

**Table 4:** Percentage of children and adolescents (2–18 years) from NHANES 2007–2010 with nutrient intakes below the EAR or above the AI based on usual intake, 100% increase in plant foods, 100% increase in protein-rich plant foods, and 100% increase in dairy foods ^1^.

Nutrient	Usual Diet (Baseline)		100% Increase in Plant Foods (Scenario 1)		100% Increase in Protein-Rich Plant Foods (Scenario 2)		100% Increase in Dairy Foods (Scenario 3)	
	UI ± SE	<EAR	UI ± SE	<EAR	UI ± SE	<EAR	UI ± SE	<EAR
		% ± SE		% ± SE		% ± SE		% ± SE
Vitamin A (μg)	618 ± 9	25.0 ± 1.1	583 ± 10	30.6 ± 1.3 *	611 ± 9	25.7 ± 1.2	825 ± 12 *	13.9 ± 0.8 *
Vitamin C (mg)	81.4 ± 1.7	21.5 ± 1.1	115 ± 2 *	12.2 ± 0.9 *	81.6 ± 1.7	21.3 ± 1.1	82.3 ± 1.9	21.1 ± 1.2
Vitamin D (μg)	6.0 ± 0.1	88.3 ± 0.6	3.6 ± 0.1 *	98.2 ± 0.3 *	5.9 ± 0.1	89.2 ± 0.5	9.9 ± 0.2 *	59.6 ± 1.1 *
Vitamin E (mg)	6.1 ± 0.1	78.1 ± 0.7	7.4 ± 0.1 *	63.5 ± 0.8 *	6.6 ± 0.1	72.8 ± 0.7	6.4 ± 0.1	75.1 ± 0.7
Folate (μg)	516 ± 6	4.4 ± 0.5	704 ± 10 *	1.8 ± 0.3 *	524 ± 7	4.1 ± 0.5	536 ± 7	3.9 ± 0.5
Magnesium (mg)	234 ± 3	35.3 ± 1.0	254 ± 3 *	30.7 ± 0.9 *	243 ± 3	33.4 ± 1.0	279 ± 3 *	26.1 ± 1.0 *
Iron (mg)	13.9 ± 0.2	2.4 ± 0.3	17.9 ± 0.2 *	1.2 ± 0.2 *	14.1 ± 0.2	2.2 ± 0.3	14.3 ± 0.2	2.0 ± 0.2
Calcium (mg)	1039 ± 12	45.1 ± 1.2	811 ± 9 *	67.5 ± 1.0 *	1026 ± 11	46.2 ± 1.1	15315 ± 18 *	20.4 ± 1.1 *
Protein (g/kg BW)	1.9 ± 0.0	1.9 ± 0.3	1.5 ± 0.0 *	5.6 ± 0.6 *	1.9 ± 0.0 *	1.9 ± 0.3	2.3 ± 0.2 *	1.0 ± 0.2 *
	UI ± SE	>AI	UI ± SE	>AI	UI ± SE	>AI	UI ± SE	>AI
		% ± SE		% ± SE		% ± SE		% ± SE
Dietary fiber (g)	13.5 ± 0.2	2.1 ± 0.2	18.2 ± 0.2 *	12.8 ± 0.7 *	14.2 ± 0.2	3.4 ± 0.3	14.0 ± 0.2	2.7 ± 0.2
Potassium (mg)	2191 ± 26	1.7 ± 0.2	2226 ± 24	2.3 ± 0.2	2222 ± 26	2.0 ± 0.2	2701 ± 28 *	9.3 ± 0.5 *
Sodium (mg)	3073 ± 32	99.5 ± 0.1	3134 ± 34	99.3 ± 0.2	3086 ± 32	99.5 ± 0.1	3368 ± 33 *	99.8 ± 0.1

^1^ Each of the modeling exercises were based on plant-based foods, protein-rich plant-based foods, and dairy foods as consumed; AI: adequate intake; EAR: Estimated average requirement; UI: usual intake ± SE: standard error; * Meaningfully different from baseline due to non-overlapping 95th percentile confidence intervals.

**Table 5:** Usual macronutrient intake from NHANES 2007–2010 compared to a 100% increase in plant foods, 100% increase in protein-rich plant foods, and 100% increase in dairy foods in adults ≥19 years ^1,2^.

Nutrient	Usual Diet (Baseline)	100% Increase in Plant Foods (Model 1)	100% Increase in Protein-Rich Plant Foods (Model 2)	100% Increase in Dairy Foods (Model 3)
Energy (kcal)	2128 ± 12	2128 ± 11	2154 ± 11	2319 ± 13 *
Carbohydrate (g)	259 ± 1	301 ± 2 *	261 ± 1	276 ± 2 *
% kcal from CHO	48.9 ± 0.2	56.9 ± 0.1 *	48.9 ± 0.1	47.9 ± 0.1 *
Protein (g)	82.1 ± 0.5	64.9 ± 0.4 *	82.1 ± 0.5	92.2 ± 0.6 *
% Kcal from protein	15.6 ± 0.1	12.1 ± 0.1 *	15.4 ± 0.1	16.1 ± 0.1 *
Total fat (g)	79.7 ± 0.6	70.0 ± 0.5 *	81.7 ± 0.6	88.8 ± 0.7 *
% Kcal from fat	32.8 ± 0.1	28.3 ± 0.1 *	33.1 ± 0.1	33.5 ± 0.1 *
Saturated fatty acids (g)	26.2 ± 0.2	20.0 ± 0.2 *	26.1 ± 0.2	31.8 ± 0.3 *
% Kcal from saturated fat	10.8 ± 0.1	8.0 ± 0.1 *	10.5 ± 0.1	11.8 ± 0.1 *
Dietary fiber (g)	16.4 ± 0.2	21.6 ± 0.3 *	17.5 ± 0.3	16.7 ± 0.2
Added Sugars (tsp eq)	18.1 ± 0.3	18.5 ± 0.3	18.1 ± 0.3	19.5 ± 0.3 *

^1^ Each of the modeling exercises were based on plant-based foods, protein-rich plant-based foods, and dairy foods as consumed; ^2^ Values are usual intake ± standard error; * Meaningfully different from baseline due to non-overlapping 95th percentile confidence intervals.

**Table 6:** Percentage of adults (19+ years) from NHANES 2007–2010 with nutrient intakes below the EAR or above the AI based on usual intake, 100% increase in plant foods, 100% increase in protein-rich plant foods, and 100% increase in dairy foods ^1^.

Nutrient	Usual Diet (Baseline)		100% Increase in Plant Foods (Model 1)		100% Increase in Protein-Rich Plant Foods (Model 2)		100% Increase in Dairy Foods (Model 3)	
	UI ± SE	<EAR	UI ± SE	<EAR	UI ± SE	<EAR	UI ± SE	<EAR
		% ± SE		% ± SE		% ± SE		% ± SE
Vitamin A (μg)	622 ± 9	46.8 ± 1.3	589 ± 9	53.3 ± 1.3 *	610 ± 9	48.6 ± 1.3	771 ± 10 *	30.7 ± 1.1 *
Vitamin C (mg)	85.3 ± 1.5	43.1 ± 1.3	113 ± 2 *	26.9 ± 1.2 *	85.6 ± 1.5	42.9 ± 1.4	85.8 ± 1.5	42.6 ± 1.4
Vitamin D (μg)	4.6 ± 0.1	95.8 ± 0.4	2.8 ± 0.1 *	99.4 ± 0.1 *	4.5 ± 0.1	96.4 ± 0.4	6.8 ± 0.1 *	81.6 ± 0.8 *
Vitamin E (mg)	7.7 ± 0.1	91.1 ± 0.7	8.7 ± 0.1 *	82.9 ± 0.9 *	8.3 ± 0.1 *	86.1 ± 1.0 *	7.9 ± 0.1	89.7 ± 0.7
Folate (μg)	544 ± 6	10.9 ± 0.7	696 ± 8 *	5.1 ± 0.5 *	556 ± 6	9.7 ± 0.7	557 ± 6	9.6 ± 0.7
Magnesium (mg)	300 ± 3	55.8 ± 1.5	330 ± 3 *	48.1 ± 1.6*	314 ± 3 *	50.8 ± 1.6	330 ± 3 *	43.7 ± 1.5 *
Iron (mg)	15.3 ± 0.1	4.3 ± 0.3	18.0 ± 0.2 *	3.2 ± 0.3 *	15.6 ± 0.1	4.0 ± 0.3	15.6 ± 0.1	4.0 ± 0.3
Calcium (mg)	972 ± 11	42.3 ± 1.2	800 ± 8 *	61.2 ± 1.2 *	960 ± 10	43.4 ± 1.2	1316 ± 17 *	22.2 ± 1.0 *
Protein (g/kg BW)	1.2 ± 0.0	3.8 ± 0.5	1.0 ± 0.0 *	16.0 ± 0.9 *	1.2 ± 0.0	3.8 ± 0.5	1.4 ± 0.0 *	2.3 ± 0.3
	UI ± SE	>AI	UI ± SE	>AI	UI ± SE	>AI	UI ± SE	>AI
		% ± SE		% ± SE		% ± SE		% ± SE
Dietary fiber (g)	16.4 ± 0.2	5.7 ± 0.5	21.6 ± 0.3 *	20.9 ± 1.0 *	17.5 ± 0.3	8.4 ± 0.6	16.7 ± 0.2	6.2 ± 0.5
Potassium (mg)	2699 ± 23	2.3 ± 0.3	2738 ± 25	2.9 ± 0.3	2746 ± 24	2.7 ± 0.3	3025 ± 27 *	6.5 ± 0.5 *
Sodium (mg)	3583 ± 23	99.8 ± 0.1	3421.0 ± 22 *	99.4 ± 0.2	3582 ± 23	99.8 ± 0.1	3827 ± 24 *	99.9 ± 0.1

^1^ Each of the modeling exercises were based on plant-based foods, protein-rich plant-based foods, and dairy foods as consumed; AI: adequate intake; EAR: Estimated average requirement; UI ± SE: usual intake ± standard error; * Meaningfully different from baseline due to non-overlapping 95th percentile confidence intervals.

## Abbreviations

The following abbreviations are used in this manuscript:

AI	Adequate Intake
DALYs	disability adjusted life years
DGA	Dietary guidelines for Americans
DRI	Dietary Reference Intakes
EAR	Estimated Average Requirement
EU	European Union
FAO	Food and Agricultural Organization
GHGEs	greenhouse gas emissions
NHANES	National Health and Nutrition Examination Survey
USDA	United States Department of Agriculture
WWEIA	What We Eat in American
